# Validity and accuracy of artificial intelligence-based dietary intake assessment methods: a systematic review

**DOI:** 10.1017/S0007114525000522

**Published:** 2025-05-14

**Authors:** Sebastián Cofre, Camila Sanchez, Gladys Quezada-Figueroa, Xaviera A. López-Cortés

**Affiliations:** 1 School of Nutrition and Dietetics, Faculty of Health Sciences, Universidad Católica del Maule, Talca, Chile; 2 PhD in Epidemiology Program, School of Public Health, Pontificia Universidad Católica de Chile, Santiago, Chile; 3 Advanced Center for Chronic Diseases, ACCDiS, Universidad de Chile and Pontificia Universidad Católica de Chile, Santiago, Chile; 4 Department of Pre-Clinical Sciences, Faculty of Medicine, Universidad Católica del Maule, Talca, Chile; 5 Department of Nutrition and Public Health, Faculty of Health and Food Sciences, Universidad del Bío-Bío, Chillán, Chile; 6 Department of Computer Sciences and Industries, Universidad Católica del Maule, Talca, Chile; 7 Centro de Innovación en Ingeniería Aplicada (CIIA), Universidad Católica del Maule, Talca, Chile

**Keywords:** Dietary assessment, Artificial intelligence, Validity, Accuracy

## Abstract

One of the most significant challenges in research related to nutritional epidemiology is the achievement of high accuracy and validity of dietary data to establish an adequate link between dietary exposure and health outcomes. Recently, the emergence of artificial intelligence (AI) in various fields has filled this gap with advanced statistical models and techniques for nutrient and food analysis. We aimed to systematically review available evidence regarding the validity and accuracy of AI-based dietary intake assessment methods (AI-DIA). In accordance with PRISMA guidelines, an exhaustive search of the EMBASE, PubMed, Scopus and Web of Science databases was conducted to identify relevant publications from their inception to 1 December 2024. Thirteen studies that met the inclusion criteria were included in this analysis. Of the studies identified, 61·5 % were conducted in preclinical settings. Likewise, 46·2 % used AI techniques based on deep learning and 15·3 % on machine learning. Correlation coefficients of over 0·7 were reported in six articles concerning the estimation of calories between the AI and traditional assessment methods. Similarly, six studies obtained a correlation above 0·7 for macronutrients. In the case of micronutrients, four studies achieved the correlation mentioned above. A moderate risk of bias was observed in 61·5 % (*n* 8) of the articles analysed, with confounding bias being the most frequently observed. AI-DIA methods are promising, reliable and valid alternatives for nutrient and food estimations. However, more research comparing different populations is needed, as well as larger sample sizes, to ensure the validity of the experimental designs.

Investigating the role of diet in health outcomes is an ongoing challenge in nutritional epidemiology and applied research^(1)^. To achieve this goal, it is necessary to get reliable data on food intake to obtain the most accurate estimates of nutrient intake and dietary patterns^(2)^. Several methods for assessing food intake have been validated in different individuals and populations. Generally, traditional methods are based on participants’ short- and medium-term memory, highlighting food records, 24-h recalls, FFQ and dietary history^(3,4)^. The choice of dietary intake method depends on the research question, study design, sample characteristics and reference timeframe^(5)^. However, an important consideration of these methods is that they are susceptible to random and systematic measurement errors that affect the reliability and accuracy of the obtained dietary information^(4)^. For example, assessment methods based on subjective evaluation, such as the 24-h recall method and FFQ, are susceptible to recall bias and researcher bias in previous discussions^(6)^. To address this limitation, Prentice *et al.* ^(7)^ conducted a study and reported that nutritional intake biomarkers could be a new approach to enhance the reliability of food records and FFQ. The cost and feasibility of these methodological approaches are barriers that researchers and health care practitioners must consider.

Rapidly evolving technologies can help to reduce the difficulties described above. Some mobile applications and web applications are becoming more prevalent in research owing to their cost-effectiveness, speed and accuracy in collecting dietary information. A review published in 2017 showed that image-assisted methods can improve the accuracy of information collection compared with conventional methods, specifically adding more detail to dietary records and being more dynamic^(8)^. Artificial intelligence (AI) has recently presented significant growth opportunities in medicine and nutrition^(9–11)^. AI-based training algorithms can support accurately predicting complex food intake interactions by integrating and organising large amounts of data^(12)^. Approaches to developing AI in nutrition include techniques such as machine learning (ML), deep learning (DL) and data mining. In turn, it can be trained in a supervised, semi-supervised or unsupervised manner^(10,13)^. These technologies are programmed to extract information from sources such as social networks, devices and mobile applications, depending on the validation context (e.g. preclinical or clinical)^(14)^. Currently, no systematic review has comprehensively and critically analysed this issue. Therefore, the present systematic review aimed to assess the validity and accuracy of AI-based dietary intake assessment methods (AI-DIA) available in the biomedical literature.

## Methods

This systematic review was designed and conducted following the Preferred Reporting Items for Systematic Reviews and Meta-analysis guidelines^(15)^. The protocol was registered in the Open Science Framework database (https://osf.io/gqw6s).

### Eligibility criteria

The PECOS (P-Population; E-Exposure, C-Comparison, O-Outcome, S-Study design) framework for the search planning was considered. The inclusion criteria were as follows: (1) human population data; (2) articles that assess dietary intake methods based on AI: 24-h recalls, FFQ, weighed food records, food records or other methods, such as image-based applications or software-based records. Each dietary intake assessment method should incorporate data processing techniques based on AI, such as DL, ML and data mining and (3) articles that report reliability properties: internal consistency, measurement error, test-retest reliability, interrater reliability, correlation coefficients and validity measures (content validity and face validity). In addition, articles report AI-metrics: accuracy, precision, regression (mean absolute error, mean squared error and root mean squared error, R^2^), ROC curve and others; (4) study designs by purpose (comparative, validation or analytical studies), by temporality (cross-sectional, prospective or retrospective studies), by researcher involvement (controlled clinical trials and quasi-experimental studies, observational) and others designs by stage of the study development (pilot studies and feasibility studies) and (5) only original articles in English language were included. The exclusion criteria were as follows: (1) studies in animal models; (2) other dietary intake assessment methods that are not based on AI; (3) study designs including ecological studies or case studies and (4) other types of articles, such as, letters to the editor, narrative reviews and conference papers.

### Search strategy and information sources

Systematic searches were performed in the Embase, PubMed, Web of Science and Scopus databases from inception to 1 December 2024 by two independent reviewers (C.S. and G.Q.). The search strategy was adapted for each database and information source according to the descriptors in the Medical Subject Headings Section section and free terms. Specifically, we used the following terms: ‘diet’, ‘dietary assessment’, ‘food intake’, ‘food records’, ‘food frequency questionnaire’, ‘24-hour recall’, ‘weighed food records’, ‘artificial intelligence’, ‘data mining’, ‘deep learning’, ‘machine learning’, ‘artificial neural networks’, ‘validity’, ‘reliability’, ‘accuracy’. All keywords were combined with Boolean operators such as OR and AND (online Supplementary 1). Records and duplicates were analysed using the Rayyan^(16)^ platform.

### Selection process and data extraction

Articles were selected, and two independent reviewers (C.S. and G.Q.) extracted their data to ensure blinding during screening. The first selection was made by assessing the relevance of the titles and abstracts identified in the search strategy and checking whether they met the eligibility criteria. In the event of disagreement during the selection process, a third reviewer (S.C.) resolved the dispute. After this stage, we analysed each selected article in the full text and removed any articles that did not meet the objective of the present review. The extraction process and analysis of the results were conducted by three researchers (C.S., G. Q. and X.A.L.C.). They entered the data into a descriptive matrix, reporting the main characteristics of the studies: (1) main author /year /country, (2) objective, (3) study design, (4) sample/ origin, (5) setting, (6) name technology (i.e. commercial or patented name), (7) dietary components evaluated, (8) traditional method of assessing dietary intake used as reference, (9) description of dietary assessment method, (10) type of AI technique used, (11) statistical method applied, (12) outcomes and (13) main findings.

### Quality assessment

Two researchers (G.Q. and S.C.) used Risk of Bias in Non-randomised Studies of Interventions, a tool developed specifically for non-randomised trials^(17)^ that assesses the risk of bias. Seven types of biases were assessed: confounding, selection of participants, classification, deviations from interventions, missing data, measurement of outcomes and reporting of results. Likewise, it assigns a rating of ‘low’, ‘moderate’, ‘serious’, ‘critical’ or ‘no information’ depending on the integration of the above domains. Additionally, a visualisation tool was used to plot the risk of bias by domain in each study.

## Results

**Figure 1.**
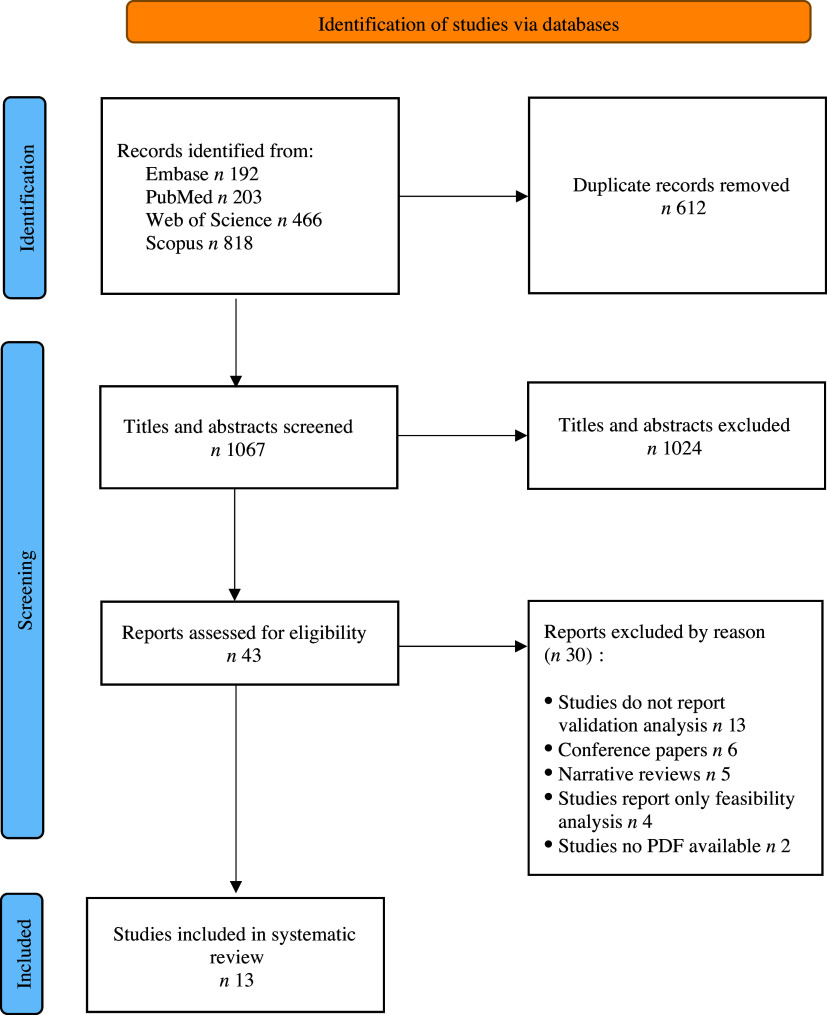
PRISMA 2020 flow diagram of process studies selection.

Our research team identified 1679 articles through a systematic search. Subsequently, 612 duplicates were removed, and 1067 titles and abstracts were screened. A total of forty-three articles were analysed, of which thirty were discarded owing to non-compliance with one or more previously defined eligibility criteria. After an exhaustive review, thirteen articles were selected for inclusion in this study (Figure 1).

### Study characteristics

In reporting the geographical distribution of the research, four studies were conducted in North America^(18–21)^, five in Asia^(22–26)^, three in Europe^(27–29)^ and only one in Africa^(30)^. By the year of publication, these ranged from 2017 to 2024, mostly concentrated in 2022 (*n* 7). Most of the designs focused on validation studies^(19,20,22–24,26,28)^ (*n* 7), followed by randomised controlled trials^(18,21)^ (*n* 2), non-randomised controlled trials^(25,30)^ (*n* 2), pilot study^(29)^ (*n* 1) and comparative studies^(27)^(*n* 1). The population sizes ranged from 36^(25)^ to 136^(21)^ participants, while the images collected varied from 576^(23)^ to 130 517^(27)^. In line with the research context, eight studies were conducted in preclinical settings, whereas four were conducted in clinical settings, as per the available information. The following AI-DIA are distinguished by their names: Food Recognition Assistance and Nudging Insights^(25,30)^, Kenooa^(18,21)^, GB HealthWatch^(19,20)^, mediPIATTO^(28)^, NutriNet^(27)^ and Automated Carbohydrate Estimation System^(22)^. The main characteristics of the AI-DIA methods are summarised in Table 1.

**Table 1.** Main characteristics of the included studies (*n* 13)

Author /year	Country	Objective	Study design	Setting	Sample/Origin	Name technology	Dietary components evaluated	Reference method used
Mezgec *et al.* (2017)^(27)^	Slovenia	To present a novel approach to the problem of food and drink image detection and recognition using a newly defined deep convolutional neural network architecture	Comparative study	Pre-clinical	520 different class food and drink contained in 225 953 images	NutriNet	Food and drink	AlexNet; GoogLeNet, ResNet
Ji *et al.* (2020)^(18)^	Canada	To assess the relative validity of an image-based dietary assessment app Keenoa – against a 3-day food diary (3DFD) and to test its usability in a sample of healthy Canadian adults	Randomised controlled trial (RCT)	Clinical	72 adults (aged 18 − 65 years)	Keenoa app	Energy, protein, carbohydrate, fat, saturated fatty acids, cholesterol, fibre, vitamins (A, B_1_, B_2_, B_12_, C, D) and minerals (Ca, Fe, Mg, P, K, Na)	3-day food diary (3DFD)
Papathanail *et al.* (2021)^(29)^	Switzerland	To evaluate the performance of our AI-based system for the estimation of energy and macronutrient intake in hospitalised patients and compare its performance with the standard clinical procedure in a geriatric acute care hospital.	Pilot study	Pre-clinical	166 meals (332 images) from 28 patients were documented	No name of AI-based technology	Energy, carbohydrate, protein, fat and fatty acids	Visual estimation by dietitians
Chotwanvirat *et al.* (2021)^(22)^	Thailand	Developed a deep learning-based system for automatic carbohydrate counting using images of Thai food taken from smartphones	Validation study	Pre-clinical	256 178 ingredients objects with measured weight for 175 food categories among 75 232 images	Automated carbohydrate estimation system (ACES)	Carbohydrate	Food weight record applied by dietitians
Kusuma *et al.* (2022)^(19)^	USA	To integrate AI machine-learning-based analytics to validate the accuracy of a mobile app against FFQ on assessing key macro- and micro-nutrients across various modern diets.	Validation study	Pre-clinical	Information from patients of colorectal cancer. 135 modern human diets were identified.	GB HealthWatch	Energy, protein, carbohydrate, fats, cholesterol, fibre, vitamins (A, B_1_, B_2_, B_12_, C, D) and minerals (Ca, Fe, Mg, P, K, Na)	30 d – FFQ
Papathanail *et al.* (2022)^(28)^	Switzerland	To propose a novel end-to-end system in the form of a smartphone application that can automatically calculate the weekly MDAS of users, based on individual images of their meals.	Validation study	Pre-clinical	Data collected with 11 024 images, along with their annotated labels and serving sizes from mediPIATTO Project	mediPIATTO	Mediterranean Diet Adherence Score (MDAS)	FFQ
Yang *et al.* (2022)^(20)^	USA	To validate the accuracy of an internet-based app against the Nutrition Data System for Research (NDSR), assessing these essential nutrients among various social-ethnic diet types.	Validation study	Pre-clinical	Information from patients of colorectal cancer. 131 social-ethnic diets were identified.	GB HealthWatch	Energy, protein, carbohydrate, fat, satured fat, cholesterol, fibre, thiamin, riboflavin, niacin, cobalamin, choline, methionine, folate, glycine, vitamins (A, C, D, E) and minerals (Zn, Ca, Mg, Fe, Na)	Data from Nutrition Data System for Research (NDSR) and app based 3-day 24-h dietary recall
Tagi *et al.* (2022)^(23)^	Japan	The accuracy of estimating leftover liquid food in hospitals using an artificial intelligence (AI)-based model was compared to that of visual estimation	Validation study	Pre-clinical	Information collected from 576 images of liquids foods delivered to a hospital.	No name of AI-based technology	Liquids, fermented milks and leftover liquid foods.	Visual estimation register conducted by dietitians
Lee *et al.* (2022)^(24)^	Taiwan	To develop an artificial intelligence model for precision nutritional analysis allows the user to enter the name and serving size of a dish to assess a total of 24 nutrients	Validation study	Pre-clinical	Available data from Health Promotion Administration, Ministry of Health and Welfare, Taiwan on 1590 recipes and 7869 food ingredients	No name of AI-based technology	Energy, water, protein, lipid fat, sugars, minerals (Ca, P, Fe, Na, Mg, K, Zn), vitamins (B_1_, B_2_, C, E, B_6_, D, B_12_), cholesterol, fibre, saturated fat.	NNHS 24-h dietary recall
Moyen *et al.* (2022)^(21)^	Canada	To evaluate the relative validity of Keenoa against a 24-h validated web-based food recall platform (ASA24) in both healthy individuals and those living with diabetes	Randomized crossover trial	Clinical	136 adults with diabetes (aged 18–70 years)	Keenoa	Energy, carbohydrate, protein, fat, fibre, Ca, Na, P and folate.	Automated self-administered 24-h dietary recall (ASA 24)
Nguyen *et al.* (2022)^(25)^	Vietnam	To assess the relative validity of FRANI (Food Recognition Assistance and Nudging Insights), a mobile artificial intelligence (AI) application for dietary assessment in adolescent females (*n* 36) aged 12–18 years in Vietnam.	Non randomised trial	Clinical	36 female adolescents (aged 12–18 years)	FRANI (Food Recognition Assistance and Nudging Insights)	Energy, protein, fat, carbohydrate, fibre, minerals (Ca, Fe, Zn), niacin, vitamins (A, B_6_, B_12_, C), riboflavin.	Food weight record
Folson *et al.* (2023)^(30)^	Ghana	To validate Food Recognition Assistance and Nudging Insights (FRANI), a mobile artificial intelligence (AI) dietary assessment application in adolescent females aged 12–18 years in Ghana	Non randomised trial	Clinical	36 females adolescents (aged 12–18 years)	FRANI (Food Recognition Assistance and Nudging Insights)	Energy, protein, fat, carbohydrate, fibre, Ca, Folate, Fe, niacin, riboflavin, thiamine, vitamins (A, B_6_, B_12_, C), Zn.	Food weight record
Tagi *et al.* (2024)^(26)^	Japan	To develop a food intake estimation system through an artificial intelligence (AI) model to estimate leftover food. The accuracy of the AI’s estimation was compared with that of visual estimation for liquid foods served to hospitalized patients.	Validation study	Clinical	300 dishes of liquid food (100 dishes of thin rice gruel, 100 of vegetable soup, 31 of fermented milk, and 69 juices)	No name of AI-based technology	Energy, protein, fat, carbohydrate.	Image visual estimation register was conducted by dietitians and direct visual estimation was carried out by nurses

### Dietary components assessed by artificial intelligence-based dietary intake assessment methods

It is worth highlighting that a significant number of methods evaluated calories, protein, fat and carbohydrates as dietary components^(18–21,24,25,29,30)^. Seven studies estimated vitamins, namely, A, D, E, C, B_1_, B_2_, B_6_ and B_12_^(18–21,24,25,30)^. In the same way, minerals such as Ca, Fe, Zn, Na, potassium, phosphorus and Mg were estimated in some studies^(21,24,25,30)^. Dietary fibre was estimated and calculated in six studies^(18–21,24,25,30)^ and water consumption in two studies^(23,24)^. Other dietary components, including food, beverages^(27)^, meals, liquids and fermented foods^(23)^, were estimated using various AI technologies, emphasising the heterogeneity of these factors in dietary intake. Only one study by Papathanail *et al.* ^(28)^ evaluated adherence to a dietary pattern, specifically the Mediterranean diet.

### Validity of artificial intelligence-based dietary intake assessment methods

A wide range of AI-DIA methods were examined and compared with the traditional methods of dietary intake assessment. Three studies utilised food weighing records, two food frequency questionnaires, two 24-hour dietary recalls, two visual estimations by dietitians, two database records with food and nutrient information and only one study employed daily food records (Table 1). Regarding the reliability of the technologies, the correlation coefficients, Pearson’s correlation and Spearman’s correlation were used. In this line, correlation coefficients between the energy estimation of AI technology and the traditional method contrasted, a variation from 0·20 in Ji *et al.* ^(18)^ study to 0·97 in the Papathanail *et al.* ^(29)^ study was observed. Moreover, the correlation coefficients for macronutrients ranged from 0·38 in the Ji *et al.* ^(18)^ study to 0·98 in the Papathanail *et al.* ^(29)^ study. Micronutrients showed a range of variation from 0·3 in the Folson *et al.* ^(30)^ article to 0·84 in the study by Kusuma *et al.* ^(19)^

Bland–Altman analyses were performed using graphical plots in 66·6 % of the AI-DIA. In the studies by Chotwanvirat *et al.* ^(22)^, Moyen *et al.* ^(21)^, Nguyen *et al.* ^(25)^ and Folson *et al.* ^(30)^, a high degree of agreement was observed for the nutrients analysed. Kappa test showed moderate agreement for micronutrients and macronutrients in the article of Nguyen *et al.* ^(25)^ Ji *et al.* ^(18)^ showed a moderate degree of agreement for fibre and certain micronutrients (vitamin A, B_1_, Mg and P), but a low degree of agreement for energy and macronutrients, also using the kappa test as a statistic. Most of the investigations employed methods for calculating the percentage of estimated differences between AI-based technology and the reference method for assessing dietary intake. These results are shown in Table 2.

**Table 2.** Summary of principal artificial intelligence (AI) and statistical techniques employed in the validation process (*n* 13)

Author /year	AI - Dietary assessment method used	Type of AI technique used	Statistical method used	Outcomes		Main findings
				Reliability	Accuracy	
Mezgec *et al.* (2017)^(27)^	The system NutriNet is based on image processing techniques applied to food photos taken by participants with their smartphones. The system detects and classifies images according to whether they are food or beverages. It then estimates dietary intake.	Deep learning (DL)	Convolutional neural network (CNN) Dataset divided into training (70 %), validation (10 %) and testing (20 %).	Not performed	86·72 %, for food and drinks classification 94·47 %, for food and drinks detection model 55 % for images captured by Parkinson’s patients	NutriNet *v*. AlexNet NutriNet *v*. GoogLeNet NutriNet *v*. ResNet Models High accuracy for classifying and detecting food and drinks. Moderate accuracy for classifying images captured by patients. NutriNet is a modification of AlexNet with 6 CNN layers.
Ji *et al.* (2020)^(18)^	Participants use Keenoa App on their smartphones to take pictures of their meals before consumption AI algorithms to recognise food items and prompts users with suggestions for identification. Consequently, users estimate serving sizes with the help of visual aids (cups, spoon). Finally, nutrient values for each food item are computed automatically by the app using data from the Canadian Nutrient platform.	Artificial intelligence algorithm (AI-Algorithm)	Pearson coefficient (*r*) Kappa score % Difference Bland–Altman test	*r* Energy: 0·2–0·49 Macronutrients: 0·38–0·51 Micronutrients: 0·42–0·47 Kappa score:< 0·20 Energy, protein, carbohydrate, fat, cholesterol, vitamins (B_2_, B_12_, C, D) and minerals (Ca, Fe, Na) Kappa score: 0·2–0·6 Fibre, Vitamin A, Vitamin B_1_, Mg, P, K.	Not performed	Kenooa-dietitian *v*. 3-Day Food diaries Moderate degree correlation in macronutrients and micronutrients. Low agreement on most nutrients, except fibre vitamin A, vitamin B_1_, Mg, P, K. Statistical differences in total energy, protein, carbohydrates, % fat, saturated fatty acids, Fe and potassium were found (*P* < 0·05). Most nutrients were within an acceptable range of agreement in the Bland–Altman analysis.
Papathanail *et al.* (2021)^(29)^	Users take a single RGB image of their meal using a smartphone camera. AI system recognizes multiple food items in each image and assigns labels to the identified food categories. Then, serving sizes are estimated directly from the image. With the information described above, the system can calculate the adherence to the Mediterranean diet, energy and macronutrients.	Deep learning (DL)	Convolutional neural network (CNN) Mean absolute error Correlation coefficient (CC)	CC Energy: 0·97 Macronutrients: 0·90–0·98	84·1 % for meals segmentation	AI-model system *v*. visual estimation by dietitians High degree of correlation in energy and macronutrients. The mean relative error was under 14 % for all nutrients. Models High accuracy for segmentation and meals estimation
Chotwanvirat *et al.* (2021)^(22)^	A Thai food image dataset was developed, containing 72 232 images of 175 food categories. Images are processed to identify and estimate the weight of food items. AI- algorithm uses factors such as object size (food), reference object area and trigger angle to refine the processing accuracy. The estimated food weights are combined with a Thai food composition database to calculate the carbohydrate content.	Deep learning (DL)	Convolutional neural network (CNN) Pearson coefficient (*r*) Lin´s concordance correlation coefficient (Rc) % Difference Bland–Altman test Dataset divided into training (80 %) and testing (20 %).	*r* Carbohydrate: 0·80 Rc Carbohydrate: 0·79	80·9 % for carbohydrate estimation of < 10 g	ACES *v*. Food weight record applied by dietitians High degree of correlation between carbohydrate estimation. High concordance in carbohydrate estimation. There is no statistical difference between both methods of estimating carbohydrate (*P* = 0·625). High degree range of agreement in the Bland–Altman analysis. Models High accuracy for carbohydrate estimation.
Kusuma *et al.* (2022)^(19)^	Dietary intakes are assessed by comparing a mobile app with FFQ. Users log meals into the app, which calculates 30 key nutrients.	Machine learning (ML)	Pearson coefficient (*r*) Standard errors (se) % Difference Bland–Altman test LOO-cv Dataset divided into training (80 %) and testing (20 %)	*r* Energy: 0·86 Macronutrients: 0·87 Micronutrients: 0·84 se Energy 1·41 Macronutrients: 1·06–5·9 Micronutrients: 1·1−2·7	AUC 0·995 for calories estimation AUC 0·921 for folate estimation AUC 0·790 for cobalamin estimation	Mobile app *v*. FFQ High degree of correlation in macronutrients and micronutrients, except Ca. Statistical differences were observed for most nutrients. Except thiamin, riboflavin, niacin, Ca. The bias (se) was greater (> 2) for fibre, vitamins A and C, Ca and Na when compared to all other nutrients. Low degree range of agreement in the Bland–Altman analysis. Models High accuracy for calories and folate estimation.
Papathanail *et al.* (2022)^(28)^	A smartphone app estimates adherence to the Mediterranean diet (MDA) through image-based recognition of foods and serving size estimation, producing a weekly score of MDA.	Deep Learning (DL)	Convolutional neural network (CNN) Mean absolute error	Mean absolute error: Mediterranean diet adherence score: 3·5 %	61·8 % for image recognition during testing set 54·5 % for serving size estimation	AI-powered system Low differences between the observed inter-rater scores. Models Moderate accuracy for imagen recognition and serving size estimation
Yang *et al.* (2022)^(20)^	Application-based internet system, where 3 d dietary intake of various ethno-social diets. The application utilises data from the USDA Food composition database and compares its calculations to NDSR. Users log dietary records, specifying the foods and portion sizes the consumed. Finally, the app analyses these records to generate nutrient profiles for each food item and aggregates the data to provide overall nutrient intake.	Machine Learning (ML)	Intraclass correlation coefficients (ICC) Standard errors (se) % Difference Bland–Altman test	ICC Energy 0·85 Macronutrients: 0·85 Micronutrients: 0·83 se Energy 1·21 Macronutrients: 1·14–1·42 Micronutrients: 1·15–2·78	AUC 0·892 for calories estimation AUC 0·904 for folate estimation AUC 0·808 for cobalamin estimation	Internet based app *v*. Nutrition Data System for Research (NDSR) High degree of correlation in macronutrients and micronutrients, except Ca. Statistical differences were observed for most nutrients. Except for carbohydrates, riboflavin, niacin, vitamin C, vitamin D, vitamin E, Ca, Mg and Fe. The bias (se) was greater (> 2) for vitamins A, C, E and Ca. Low degree range of agreement in the Bland–Altman analysis. Models High accuracy for calories, folate and cobalamin estimation.
Tagi *et al.* (2022)^(23)^	Liquid foods were photographed on hospital trays, including a variety of items such as thin rice gruel (staple food), fermented milk and peach juice (side dishes). A one-class detection model identified liquid food as the foreground and everything else as the background. AI estimated the percentage of food consumed or leftover based on image analysis. Then system calculated nutritional intake indirectly by quantifying the remaining portion	Deep Learning (DL)	Convolutional neural network (CNN) Mean absolute error Bland–Altman test	Mean absolute error: Fermented milk 77 % Peach juice 67 % Total food 18 %	99 % for thin rice gruel leftover estimation 63 % for fermented milk leftover estimation 25 % for peach juice leftover estimation 85 % for total leftover foods estimation	Algorithm *v*. visual estimation register by dietitians Moderate degree range of agreement in the Bland–Altman analysis for fermented milk and peach juice Low degree range of agreement in the Bland–Altman analysis for thin rice gruel and total foods. Models High accuracy for thin rice gruel and total foods quantification. Moderate accuracy for fermented milk and peach juice quantification.
Lee *et al.* (2022)^(24)^	Dietary intake is calculated by entering dish names and serving sizes into a precision nutrient analysis system. Recipes and portion sizes are extracted via semantic AI analysis.	Natural language processing (NLP)	% Difference	Discrepancy error range Macronutrients: < 3 %, except sugars < 10 % Micronutrients: < 6 %, except VitaminD2D3 < 10 %	Not explained	Algorithms *v*. NNHS 24-hour dietary recall Low level of discrepancy in macronutrients and micronutrients estimation, except for sugars and vitamin D.
Moyen *et al.* (2022)^(21)^	Keenoa is an image-assisted food-tracking mobile app that uses artificial intelligence for food recognition and portion size quantification.	Artificial intelligence algorithm (AI-A)	Spearman correlation coefficient (CC) Weighted Cohen Kappa Bland–Altman test	CC Stratified by sex Woman Energy: 0·71 Macronutrients: 0·42–0·84 Micronutrients: 0·30–1 Men Energy: 0·85 Macronutrients: 0·68–1·0 Micronutrients: 0·61–1·0 Weighted Cohen Kappa Energy: 0·45 Macronutrients: 0·29–0·52 Micronutrients: 0·31–0·49	Not performed	Keenoa *v*. ASA 24 In women, moderate degree correlation for most macronutrients micronutrients and energy. In men, moderate degree correlation for most macronutrients and micronutrients. Except in energy correlation. No statistical differences were found in means for most nutrients. Except, fibre (*P* < 0·001), Fe (p 0·002), Na (*P* < 0·001) High degree range of agreement in the Bland–Altman analysis. Nevertheless, Cohen kappa´s cross-classification of the majority of nutrients was relatively moderate.
Nguyen et al (2022)^(25)^	The FRANI app used an AI-based food recognition algorithm to classify food items from images, and it also allows estimating portion size. Additionally, the users could manually confirm, or correct food classifications returned by the AI algorithm and adjust portion sizes using a database available for this purpose. Finally, the dietary intake data are converted into nutrient profiles.	Artificial intelligence algorithm (AI-A)	Concordance correlation coefficients (CCC) Test equivalence Bland–Altman test	CCC Energy: 0·63 Macronutrients: 0·63–0·74 Micronutrients: 0·67–0·81	Not performed	FRANI *v*. Food weight record Equivalence for 10 % bound for energy, protein, fat and 4 micronutrients (Fe, riboflavin, vitamin B_6_ and Zn). 15 % and 20 % bounds for carbohydrate, Ca, vitamin C, thiamin, niacin and folate. Moderate degree correlation in energy, macronutrients and micronutrients. Except Vitamin C. High degree range of agreement in the Bland–Altman analysis for energy, macronutrients, micronutrients.
Folson *et al.* (2023)^(30)^	The FRANI app used an AI-based food recognition algorithm to classify food items from images, and it also allows estimating portion size. Additionally, the users could manually confirm, or correct food classifications returned by the AI algorithm and adjust portion sizes using a database available for this purpose. Finally, the dietary intake data are converted into nutrient profiles.	Artificial intelligence algorithm (AI-A)	Concordance correlation coefficients (CCC) Test equivalence Bland–Altman test	CCC Energy: 0·36 Macronutrients: 0·42–0·54 Micronutrients: 0·3–0·68	Not performed	FRANI *v*. Food weight record Equivalence for 10 % bound for energy intake, 15 % for five nutrients (Fe, Zn, folate, niacin and vitamin B_6_), 20 % for protein, Ca, riboflavin and thiamine intakes. Low degree correlation in energy and macronutrients Moderate degree correlation in micronutrients. High degree range of agreement in the Bland–Altman analysis for energy, Fe, and vitamin A.
Tagi *et al.* (2024)^(26)^	Images of liquid foods are taken after meals using a mobile device. The AI processes these images to identify the area, name and amount of leftover food. Then nutritional intake is calculated by multiplying the nutrient composition of the provided food with the proportion of food consumed.	Deep learning (DL)	Spearman correlation coefficients (CC) Paired *t* test Bland–Altman test Root mean square error (RMSE) Mean absolute error (MAE)	CC Energy: 0·89 Macronutrients: 0·89–0·94 RMSE Energy: 8·12 kcal Macronutrients: 14·6 mg–1·95 g MAE Overall 6·1 %	Energy 88 % Protein 91 % Fat 93 % Carbohydrate 88 %	Algorithm *v*. Visual estimation register conducted by dietitians High degree range of agreement in the Bland–Altman for all nutrients. Nevertheless, the limits of agreement for the Bland–Altman plots were wider for the AI and image visual estimation than for the direct visual estimation models High accuracy for energy and macronutrient estimations was observed.

### Artificial intelligence techniques applied in artificial intelligence-based dietary intake assessment methods

Regarding the types of AI techniques employed by the various technologies under review, DL was identified as a method of processing dietary data in five studies^(22,23,27–29)^. In two articles, the use of ML was reported^(19,20)^. Conversely, four authors only reported the use of an AI-algorithm and did not provide information about the type of analysis technique used for its validation^(18,21,25,30)^. Chotwanvirat *et al.* ^(22)^ employed DL techniques for food recognition and food weight estimation, particularly a neural network-based regression model. For his part, Lee *et al.* ^(24)^ used the technique known as natural language processing to analyse their data. In turn, two studies described the stages of training, validation and testing of the AI models. Mezgec *et al.* ^(27)^ for the DL model divided the dataset into 70 % training, 10 % validation and 20 % testing. In the case of Kusuma *et al.* ^(19)^, in their ML model, data processing was divided into two stages: 80 % training and 20 % testing. (Table 2). Also, Tagi *et al.* ^(26)^ used convolutional neural networks as the technique and training of their model to analyze liquids estimation from images.

### Accuracy of artificial intelligence-based dietary intake assessment methods

The accuracy of the AI models was estimated in seven investigations. Mezgec *et al.* ^(27)^ obtained 94·47 % in a food and drink detection model for a set of images captured by the study participants. Papathanail *et al.* ^(29)^ achieved 61·8 % in their model for recognising meal images and 54·5 % for serving size estimation. In another study by his authorship, he achieved 84·1 % meal segmentation in the model^(28)^. In contrast, Tagi *et al.* ^(23)^ evaluated the accuracy of their algorithm for estimating leftover food and obtained an accuracy of 99 % for thin rice gruel, 63 % for fermented dairy, 25 % for peach juice and 85 % for all foods.

Kusuma *et al.* ^(19)^ developed a predictive model to account for the observed differences between nutrient estimations from the application and the FFQ. An AUC of 0·995 was obtained to explain the differences in calorie estimates based on carbohydrate and protein differences. In a related study, Yang *et al.* ^(20)^ reported an AUC of 0·91 to account for differences in estimated calories between an internet-based application and a national database recording participants’ intake information (Table 2).

### Risk of bias

**Figure 2.**
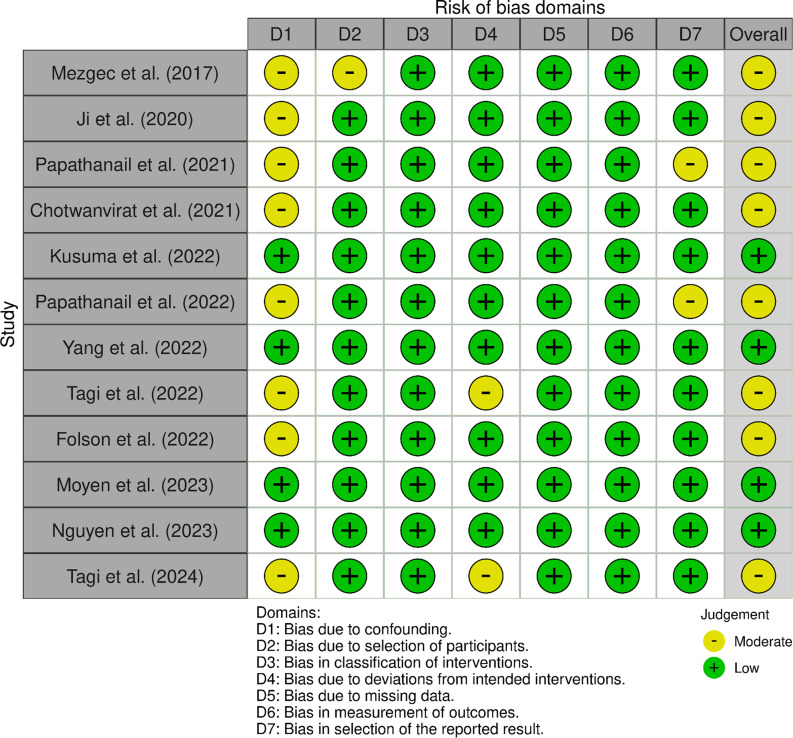
Risk of bias (ROB) in selected studies.

A quality assessment using the ROBINS tool revealed that 58·3 % of the studies were at moderate risk of bias (online Supplementary 2). In contrast, 25 % showed a low risk of bias, most of which were experimental. Overall, confounding bias was the most frequently reported in the present analysis for moderate risk assessment. Intervention intention bias, missing data bias and outcome measurement bias at the judgement of the reviewers were presented with a lower risk of bias. These results are shown in Figure 2.

## Discussion

This is the first systematic review to examine the validity and accuracy of dietary intake assessment methods that employ AI techniques in dietary information processing. The selected studies suggest the promising potential of AI-DIA, indicating a possible evolution in comparison with traditional dietary assessment methods. However, further research is needed to confirm and quantify its actual impact.

Consequently, the increasing number of published articles demonstrates a growing interest in research on the use of technologies based on AI, particularly in the most recent period, between 2022 and 2024. Furthermore, there is a high concentration of research in North America^(18–21)^ and Asia^(22–25)^, which poses a challenge to the generalisability of results. Our research findings indicate that traditional methods of intake assessment, particularly food weight record and 24-h dietary recall, are more prevalent than other methods to contrast the validity of AI-DIA^(20–22,24–26,30)^.

Our systematic review highlights the analysis of the main AI and statistical techniques used in the validation of dietary intake assessment methods, reporting that most of the studies focus on app uses that have behind them the use of AI, either focused on ML^(19,20)^ or DL^(22,23,27–29)^. The implementation of advanced algorithms, including convolutional neural networks and ML, which have revolutionised the accuracy and efficiency in estimating dietary and nutritional intake from images captured by mobile devices^(19,27)^.Others studies reported in detail procedures to detect and classify images of the evaluated algorithms, for instance in the studies Mezgec *et al.* ^(27)^ and Yang *et al.* ^(20)^ showed high levels of accuracy in food classification and detection, comparable or superior to traditional methods. These findings are consistent with those discussed in the systematic review conducted by Ho *et al.* ^(31)^, where he points out that app-based photographic capture methods are highly accurate, even superior to dietician-directed methods, allowing for adequate classification and quantification of food portion sizes.

Concerning to the procedures necessary for validation of AI-DIA methods, our research suggests that this can be based on classifying foods, quantifying portions and estimating calories, macronutrients and micronutrients. For calorie estimation, four studies showed correlation coefficients (CC) higher than 0·8, with the research by Papathanail *et al.* ^(29)^ standing out, whose identification system achieved a CC of 0·97 compared to the visual estimation developed by dieticians. In this research, an adequate amount of food components and plate types (*n* 4) seems to be beneficial for model training, increasing accuracy, the authors conclude. In terms of macronutrient estimation, four studies reported CC above 0·8, showing heterogeneity in the indices, for instance, Moyen *et al.* ^(21)^ reported a higher level of correlation in the estimation of protein and carbohydrates in men than in women. Furthermore, the findings of Kusuma *et al.* ^(19)^ indicate a high CC (0·85–0·87) for all macronutrients, when the mobile app was compared with the food frequency questionnaire, using ML techniques for the analysis. Similarly, Yang *et al.* ^(20)^ compared an internet-based application and the USDA Nutrition Data System for Research showing a CC 0·85 and thus confirming the validity of ML-based technology. These findings indicate that the use of AI-DIA has an adequate correlation and reliability for the estimation of macronutrients. In the case of micronutrients, there is a high variation in the estimates, making it difficult to generalise results. To demonstrate this issue, Kusuma *et al.* ^(19)^ reported a CC of 0·84, highlighting the estimates of vitamin D (0·90), Fe (0·88) and niacin (0·88). In contrast, Ji *et al.* ^(18)^ reported low CC (< 0·20) especially in micronutrients such as vitamin B_12_, vitamin C, vitamin D, Ca, Mg, potassium and Na when they compared Kenooa-dietitian *v*. 3-day food diary records. Likewise, Folson *et al.* ^(30)^ obtained low CC niacin (0·42), riboflavin (0·51), vitamin A (0·47) and vitamin C (0·3) when they compared FRANI-app *v*. Weighed Records. These differences emphasise the difficulty in estimating micronutrients from the AI-DIA compared to the traditional method, posing challenges in standardising methods that allow contrast of technologies, and the use of homogeneous databases, even knowing that there are variations between populations in the quantification of intakes. Capling *et al.* ^(32)^ previously discussed this problem, pointing out that dietary intake assessment methods that use long recall periods, namely, FFQ, may have low accuracy for micronutrient assessment and have low CC. Another issue in micronutrient analysis is the ability to determine the amount of minerals and vitamins in various food sources, which depends on the quality of information in national databases containing the chemical composition of foods.

Another method employed in most of the studies was the use of Bland–Altman plots, which demonstrated a considerable degree of heterogeneity in the analyses. To illustrate this point, in five articles, a high-moderate degree of agreement^(18,21,22,25,30)^ was observed for the nutrients reported; however, in three, a low degree of agreement^(19,20,23)^ was observed.

Concerning the AI techniques used by the AI-DIA, there is a wide predominance of DL, highlighting the CNN analysis rules as the main learning architecture. Chotwanvirat *et al.* ^(22)^ used CNN to process images of carbohydrate-based ingredients from Thai cuisine, comparing the results with food weight recording performed by dietitians. For its part, Papathanail *et al.* ^(28)^ developed a CNN-based system for recognising foods and calculating Mediterranean diet adherence scores from food photos, compared to a FFQ. Tagi *et al.* ^(23)^ used a multitask CNN to classify the names of liquid foods and estimate the leftover liquid food, in addition the model considered calorie-volume estimation. The research described above demonstrates the effectiveness of DL for food image recognition, which is achieved through computational models composed of multiple processing layers and that are trained by input based on image sets. Specifically, CNN layers contain learnable filters that respond to features in the input data, and fully connected layers compose output data from other layers to obtain higher level learning from them^(33)^.

A relevant discussion to note is that food record methods and 24-h dietary recall were the most employed reference methods to validate and contrast with AI-DIA. In particular, the food weighing method has been shown to be a reliable method (gold standard) to compare the different AI technologies examined, provided that trained dietitians are available to perform the measurements. Despite its notable advantages, Ortega *et al.* ^(34)^ argue that this method requires time, the possible induction of modifications in the diet of the subjects analysed or difficulties in describing the foods and/or portions consumed when it is self-applied.

The systematic review revealed significant variability in the quality of included studies, with 61·5 % presenting a moderate risk of bias and 38·5 % low risk, according to the Risk of Bias in Non-randomised Studies of Interventions tool. The most common biases were confounding and participant selection, suggesting the presence of uncontrolled variables that could influence results^(35,36)^. These findings are consistent with previous studies that have identified the presence of biases in AI-based research, particularly in the context of dietary and health assessment^(37)^.

Randomised controlled trials are considered the gold standard for intervention validation, offering robust evidence on the efficacy and accuracy of AI technologies compared to traditional methods^(38)^. For example, Moyen *et al.* ^(21)^ led a randomised crossover design to evaluate the validity of an AI-assisted diet application against a web-based food recall method, demonstrating the robustness of randomised controlled trials for validating AI technologies. However, most of the reviewed studies employed non-randomised designs, limiting the assessment of causality and clinical effectiveness.

The implementation of randomised controlled trials to validate AI technologies faces significant challenges, including high costs, logistical complexity, the need for large samples and variability in dietary intake. Additionally, result generalisability, rapid technological evolution and ethical considerations present additional challenges. These challenges have been documented in studies analysing the feasibility of technology-based interventions to improve nutrition, like the Nudging for Good project^(39)^.

### Strengths and limitations

Our review is the first with focus on the validity and accuracy of AI-based dietary assessment methods, underlining its emphasis on the wide variety of designs and technologies currently under development in research. In this regard, a high percentage of mobile applications currently under development and validation, both in preclinical and clinical contexts, was observed. During the protocol design phase, rigorous inclusion criteria were established, therefore all the selected articles present some AI-DIA that are contrasted with a traditional method for the evaluation of dietary intake. Moreover, we performed an exhaustive comparison of the designs of the included studies, as well as an analysis of the quality of the evidence using a standardised tool, reporting a critical look at the risk of bias of the studies.

Some limitations are mainly focused on three aspects. First, the heterogeneity of contrast methods for the evaluation of dietary intake prevented us from developing a meta-analysis to summarise the pooled effect of the investigations. This issue is problematic, especially if the studies in a meta-analysis differ significantly because the generalisability of the pooled estimate could be questionable, losing clinical applicability^(40)^. Second, despite the exhaustive analysis in each article, some did not report the AI technique used, whether ML or DL, they only noted the use of an AI algorithm. Wang *et al.* ^(41)^ recently led a guide for the development, validation and evaluation of AI-based algorithms where it is recommended to explain in detail the development of a new algorithm to ensure the transparency and reproducibility of the research. It is emphasised to choose appropriate approaches to algorithm development, such as inclusion of coding, model-based rules and explicit ML techniques employed^(41)^. Another salient limitation of the present review is the potential for its included studies to lack representativeness with respect to diverse cultural and demographic contexts. AI-based tools could have varied performance in different cultures due to differences in diets, eating habits and food availability. Future studies should focus on cross-cultural validation of AI-DIA to ensure its applicability and accuracy in diverse populations.

### Future directions

From the point of view of research designs, it is recommended that standardised protocols be developed, multicentre collaborations be encouraged, technology assessment frameworks be updated and adaptive designs be explored. Adaptive designs can make clinical trials more flexible by using the results accumulated in the trial to modify the course of the trial according to previously defined rules^(42)^. These strategies aim to improve the validity and reliability of AI- DIA, ensuring their accuracy, safety and ethical acceptability.

In addition, we suggest encouraging the use of one or more traditional methods of dietary intake to allow for a greater variety of methods and homogenise future statistical analyses between studies. In this context, the methods of dietary record and weighed food are appropriate for comparing AI technologies, according to our analysis.

Finally, as AI technologies become more prevalent in dietary intake assessment, ethical frameworks and regulatory standards will need to be established to govern their use^(43)^. Ensuring that AI-DIA are developed with the inclusion of ethical frameworks in mind will avoid potential biases that could arise from biased training data^(43)^. Collaborations with local researchers and academic partners have the potential to enhance the cultural relevance of AI dietary solutions, fostering trust and stimulating effective utilisation in diverse communities.

### Conclusions

Validity and accuracy are fundamental properties of any method that assesses nutrient or food intake in individuals. This systematic review critically analysed all the available evidence, highlighting as findings that AI-DIA are presented as reliable and valid methods to determine the amount of energy and macronutrients of individuals. Although the validity for micronutrient determination is moderate to low due to the variability of information contained in the sources and resources used as input in the ML or DL models. A relevant challenge is the design of randomised clinical trials to evaluate the efficacy of AI-DIA, as there is a moderate risk of bias in the designs included in the present study.

## Supporting information

Cofre et al. supplementary material Cofre et al. supplementary material

## Supplementary material

For supplementary material/s referred to in this article, please visit https://doi.org/10.1017/S0007114525000522
