# Supplementary material for: Validity and accuracy of artificial intelligence-based dietary intake assessment methods: a systematic review
Source: Br J Nutr. 2025 Apr 10;133(9):1241–53. doi: 10.1017/S0007114525000522 (PMC12229984; doi:10.1017/S0007114525000522)
Supplement: Cofre et al. supplementary material [file S0007114525000522sup001.docx]

**Supplementary 1**

**Search pattern by database**

Date: 01-12-2024

| PUBMED |
| --- |
| (((((((("diet"[Title/Abstract]) OR ("dietary assessment"[Title/Abstract])) OR ("food intake"[Title/Abstract])) OR ("food records"[Title/Abstract])) OR ("food frequency questionnaire"[Title/Abstract])) OR ("24 hour recall"[Title/Abstract])) OR ("weighed food records"[Title/Abstract])) AND ((((("artificial intelligence"[Title/Abstract]) OR ("data mining"[Title/Abstract])) OR ("deep learning"[Title/Abstract])) OR ("machine learning"[Title/Abstract])) OR ("artificial neural network"[Title/Abstract]))) AND (((("validity"[Title/Abstract]) OR ("reliability"[Title/Abstract])) OR ("feasibility"[Title/Abstract])) OR ("accuracy"[Title/Abstract])) |

| EMBASE |
| --- |
| #1 diet:ti,ab,kw OR 'nutritional assessment':ti,ab,kw OR 'food record':ti,ab,kw OR 'food frequency questionnaire':ti,ab,kw OR '24 hour recall':ti,ab,kw OR 'weighed food records':ti,ab,kw  #2 'artificial intelligence':ti,ab,kw OR 'data mining':ti,ab,kw OR 'deep learning':ti,ab,kw OR 'machine learning':ti,ab,kw OR 'artificial neural network':ti,ab,kw  #3 'validity':ti,ab,kw OR 'reliability':ti,ab,kw OR 'feasibility':ti,ab,kw OR accuracy:ti,ab,kw  #1 AND #2 AND #3 |

| SCOPUS |
| --- |
| ( ( TITLE-ABS-KEY ( "diet" ) OR TITLE-ABS-KEY ( "dietary assessment" ) OR TITLE-ABS-KEY ( "food intake" ) OR TITLE-ABS-KEY ( "food frequency questionnaire" ) OR TITLE-ABS-KEY ( "24 hour recall" ) OR TITLE-ABS-KEY ( "weighed food records" ) ) ) AND ( ( TITLE-ABS-KEY ( "artificial intelligence" ) OR TITLE-ABS-KEY ( "data mining" ) OR TITLE-ABS-KEY ( "deep learning" ) OR TITLE-ABS-KEY ( "machine learning" ) OR TITLE-ABS-KEY ( "artificial neural network" ) ) ) AND ( ( TITLE-ABS-KEY ( "reliability" ) OR TITLE-ABS-KEY ( "validity" ) OR TITLE-ABS-KEY ( "feasibility" ) OR TITLE-ABS-KEY ( "accuracy" ) ) ) |

| Web of Science |
| --- |
| #1 diet (All Fields) or dietary assessment (All Fields) or food intake (All Fields) or food frequency questionnaire (All Fields) or 24 hour recall (All Fields) or weighed food records (All Fields)  #2 artificial intelligence (All Fields) or data mining (All Fields) or deep learning (All Fields) or machine learning (All Fields) or artificial neural network (All Fields)  #3 (((ALL=("reliability" )) OR ALL=("validity")) OR ALL=("feasibility")) OR ALL=("accuracy")  #1 AND #2 AND #3 |

**Supplementary 2**

**Risk of bias of nonrandomized studies using ROBINS-I tool**

| **Study** | **D1** | **D2** | **D3** | **D4** | **D5** | **D6** | **D7** | **Overall** |
| --- | --- | --- | --- | --- | --- | --- | --- | --- |
| Mezgec et al. (2017) | Moderate | Moderate | Low | Low | Low | Low | Low | Moderate |
| Ji et al. (2020) | Moderate | Low | Low | Low | Low | Low | Low | Moderate |
| Papathanail et al. (2021) | Moderate | Low | Low | Low | Low | Low | Moderate | Moderate |
| Chotwanvirat et al. (2021) | Moderate | Low | Low | Low | Low | Low | Low | Moderate |
| Kusuma et al. (2022) | Low | Low | Low | Low | Low | Low | Low | Low |
| Papathanail et al. (2022) | Moderate | Low | Low | Low | Low | Low | Moderate | Moderate |
| Yang et al. (2022) | Low | Low | Low | Low | Low | Low | Low | Low |
| Tagi et al. (2022) | Moderate | Low | Low | Moderate | Low | Low | Low | Moderate |
| Lee et al. (2022) | No information | No information | No information | No information | No information | No information | Low | No information |
| Folson et al. (2023) | Moderate | Low | Low | Low | Low | Low | Low | Moderate |
| Moyen et al. (2023) | Low | Low | Low | Low | Low | Low | Low | Low |
| Nguyen et al. (2023) | Low | Low | Low | Low | Low | Low | Low | Low |
| Tagi et al. (2024) | Moderate | Low | Low | Moderate | Low | Low | Low | Moderate |

**Overall risk is classified as:**

Low: If all domains have a low risk.

Moderate: If at least one domain has a moderate risk.

No information: If information is missing to assess at least one key domain.
